# Serum Butyrylcholinesterase Activity: A Biomarker for Parkinson's Disease and Related Dementia

**DOI:** 10.1155/2017/1524107

**Published:** 2017-08-03

**Authors:** Mei-Xue Dong, Xiao-Min Xu, Ling Hu, Yang Liu, Yuan-Jun Huang, You-Dong Wei

**Affiliations:** ^1^Department of Neurology, The First Affiliated Hospital of Chongqing Medical University, Chongqing, China; ^2^Department of Neurology, Chongqing Fifth People's Hospital, Chongqing, China

## Abstract

**Objective:**

This study aim to determine changes of serum butyrylcholinesterase (BChE) activity in PD patients and related dementia.

**Patients and Methods:**

Consecutive PD patients and healthy controls were included and clinical data were collected. Fast serum BChE activity was determined and compared between healthy controls and PD patients. Independent risk factors were performed for BChE activity, PD, and related dementia. The relationship between BChE activity and disease severity was also evaluated. Receiver operating characteristic (ROC) curves were obtained to explore serum BChE activity in distinguishing PD patients and related dementia.

**Results:**

Serum BChE activity mainly independently correlated with gender, albumin, triglyceride, body mass index, and PD. Serum BChE activity decreased in PD patients compared with healthy controls. Based on the ROC curve, the optimal cut-off point was 6864.08 IU/L for distinguishing PD patients, and the sensitivity and specificity values were 61.8% and 72.1%. It inversely correlated with Unified Parkinson's Disease Rating Scale score. BChE activity decreased in PD-related dementia compared with those without dementia. The sensitivity and specificity values were 70.6% and 76.3%, respectively, with an optimal cut-off point of 6550.00 IU/L.

**Conclusions:**

Serum BChE activity can be regarded as a biomarker for PD and related dementia.

## 1. Introduction

Parkinson's disease (PD), the second most common neurodegenerative disease following Alzheimer's disease, afflicts about 1% of the elderly over 60 years old and 3% of those over 80 years old [1]. Although extensive studies have been performed to identify PD biomarkers, no validated biomarkers have been found to date and the diagnosis remains based primarily on clinical symptoms. PD is a complicated interplay of genetics and the environment, typically presenting as death of dopaminergic neurons in the substantia nigra pars compacta [2]. However, it also involves alterations of neurotransmitters other than dopamine and regions of the nervous system outside the substantia nigra [2]. Those various pathological changes result in many other nonmotor symptoms, including olfactory dysfunction, cognitive impairment, depression, anxiety, sleep disorders, autonomic dysfunction, pain, and fatigue.

Cognitive impairment is common in PD patients and is associated with age, disease duration, and disease severity. It can also develop in PD patients following treatment with anticholinergic drugs, such as benzhexol and benztropine. More than 80% of PD patients with cognitive impairment develop dementia over a long-term disease duration [3]. PD patients with dementia bring particular challenges to patient management, which further aggravates functional impairments caused by motor symptoms [4]. Patients with dementia have greater requirements of families and caregivers in terms of activities of daily living, resulting in a social-psychological strain for both PD patients and their families. Early dementia treatment would be more effective [5]. Hence, there is an urgent need to identify potential biomarkers for Parkinson's disease and related dementia.

With exception of changes in dopaminergic neurons in the nigrostriatal pathway, a former PET study showed that PD patients with dementia also had suffered from severe cholinergic deficits all over the brain [6]. Acetylcholine (Ach) is the main parasympathetic neurotransmitter released by cholinergic neurons. It is a key contributor to stress and enhances neuronal excitability. In addition, it also potently modulates the classical immune response by activating *α*7 nicotinic Ach receptor on the surface of leukocytes in the so-called “cholinergic anti-inflammatory response” [7]. The cholinergic system is involved in many physiological functions of the central nervous system, and Ach can be hydrolyzed by acetylcholinesterase (AChE) and butyrylcholinesterase (BChE), two independent enzymes in the circulation system. Serum BChE is mainly synthesized in the liver and can be clinically used as a marker of organophosphate poisoning [8]. However, increasing evidence suggests that BChE activity is also associated with disease severity and can be used to predict prognosis in many diseases [7]. Although AChE activity reportedly decreases in Parkinson's disease [9], the relationship between BChE activity and Parkinson's disease had not been clearly performed.

Serum is a relatively noninvasive and reliable clinical specimen. The present study aimed to determine changes of serum BChE activity in PD patients and related dementia.

## 2. Materials and Methods

### 2.1. Patients

A total of 84 consecutive patients with Parkinson's disease were included in accordance with the following inclusion criteria from the Department of Neurology of the First Affiliated Hospital of Chongqing Medical University from April 2015 to December 2016. In all, eight patients were excluded per the exclusion criteria. The inclusion criteria were as follows: (i) Parkinson's disease diagnosed according to the recommendation from EFNS and MDS-ES [10]; (ii) patients treated without any drugs with a known effect on cholinesterase (ChE) activity (including anticholinergic drugs benzhexol, benztropine, atropine, anisodarnine, and scopolamine; ChE inhibitors donepezil, galantamine, and rivastigmine; statins; acetylsalicylic acid; isosorbide-based drugs; fluoxetine, sertraline, and amitriptyline; benzodiazepine derivates; itopride; chlordiazepoxide; histamine 2 receptor antagonist; or Ginkgo extract) [11]. Exclusion criteria were as follows: (i) secondary parkinsonian disorders or parkinsonism-plus syndrome; (ii) coexistence of severe systemic diseases (e.g., tumor, chronic heart failure, chronic obstructive pulmonary disease, hepatitis, and nephritis) or infectious diseases at the time of enrolment; (iii) history of stroke, brain surgery or injury, Alzheimer's disease, motor neuron disease, or other central nervous system diseases [12, 13].

### 2.2. Healthy Controls

A total of 61 healthy controls were included at the same time from the Department of Physical Examination. The controls were without history of stroke, brain surgery or injury, neurodegenerative disease, or infectious disease. This study was approved by the ethics committee of the First Affiliated Hospital of Chongqing Medical University and performed in accordance with ethical principles. All subjects provided written informed consent prior to inclusion in this study.

### 2.3. Clinical Characteristics

Clinical data from patients and healthy controls were collected. Fast serum samples were obtained by centrifugation at 2000 ×g for 10 min at 4°C after being collected at 6:00 am by puncture of the median cubital vein. Then, levels of albumin, alanine transaminase (ALT), aspartate aminotransferase (AST), total cholesterol (TC), triglyceride (TG), and BChE activity were measured soon using a Cobas Integra 400 plus automatic biochemical analyser with matched reagent kits (Roche, Basel, Switzerland). Serum BChE activity was determined using Cobas Integra Cholinesterase kit (20737380; Roche) and butyrylthiocholine served as the substrate. Other laboratory benchmarks were quantified per manufacture specifications.

### 2.4. Disease Assessment Scale

Unified Parkinson's disease rating scale (UPDRS), modified Hoehn-Yahr staging scale, and Mini-Mental State Examination (MMSE) scores were assessed at the time blood specimens were collected. Total UPDRS scores were used to assess disease severity of PD. Modified Hoehn-Yahr staging scale scores were used to group PD patients into early stage (<2.5) or advanced stage (≥2.5) [14]. MMSE scores were used to detect cognitive impairment in PD patients, and three different cut-off points were used to determine dementia according to patient education level [15]. All scales were independently assessed by two observers.

### 2.5. Statistical Methods

Statistical analyses were performed using a commercially available software package (IBM SPSS version 22.0, Armonk, NY, USA). Some statistical diagrams were produced using GraphPad Prism 5 (Version 5.01, GraphPad Software, La Jolla, CA, USA). Clinical characteristics were compared between healthy controls and PD patients, with or without dementia, using Pearson *χ*^2^-tests or Fisher exact tests for categorical data and Student's *t*-tests for continuous data. Categorical data were exhibited as absolute numbers and percentage (%), while continuous data were exhibited as mean values ± standard deviation. Univariate and multivariate linear regression analyses were performed to determine independent correlation factors for BChE activity. Univariate and multivariate binary logistic regression analyses were performed to determine independent correlation factors for PD and related dementia. Pearson correlation coefficients were obtained and scatter diagrams were plotted to explore the relationships between BChE activity and UPDRS scores or MMSE scores. To assess the reliability of serum BChE activity in discriminating PD or related dementia, receiver operating characteristic (ROC) curves were plotted and the areas under the curve (AUCs) were estimated. Based on ROC curves, the sensitivity and specificity values were calculated according to optimal cut-off points. Tests were two-tailed and significance levels were chosen as *P* < 0.05.

## 3. Results

### 3.1. Clinical Characteristics of PD Patients and Healthy Controls

**Figure 1:**
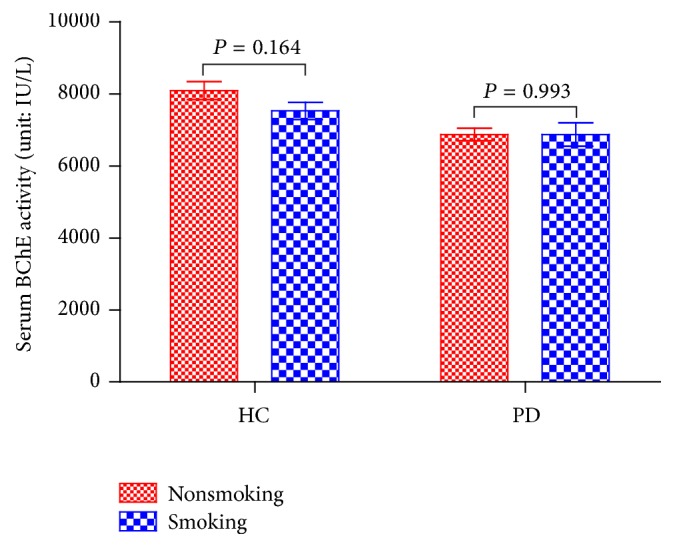
*Mean serum BChE activities of different subgroups based on smoking history*. No significant difference can be found between smoking and nonsmoking patients in the two groups (*P* > 0.05). BChE, butyrylcholinesterase; HC, healthy control; PD, Parkinson's disease.

**Figure 2:**
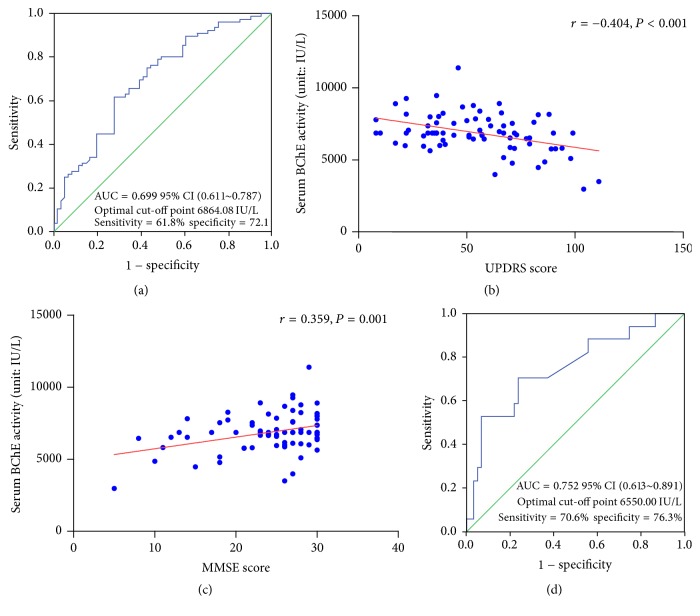
*(a) ROC curve of serum BChE activities for detecting PD patients. (b) A linear correlation is observed between BChE activities and UPDRS scores. (c) A linear correlation is observed between BChE activities and MMSE scores. (d) ROC curve of serum BChE activities for detecting PD patients with dementia*. ROC, receiver operating characteristic; BChE, butyrylcholinesterase; PD, Parkinson's disease; AUC, area under the curve; CI, confidence interval; UPDRS, Unified Parkinson's Disease Rating Scale; MMSE, Mini-Mental State Examination.

All included patients had been diagnosed prior to the study and were treated with dopamine analogues or dopamine receptor agonists at the time of enrolment. Clinical characteristics of PD patients and healthy controls are presented in Table 1. There were no significant differences between these two groups in age, gender, or any other characteristics. Serum BChE activity in PD patients were lower than in healthy controls. PD patients had a slightly lower percentage of smoking history than healthy controls. According to the linear regression analyses presented in Table 2, gender, albumin, TG, BMI, and PD were all independent correlation factors for serum BChE activity while smoking history was not. For PD, only smoking history and BChE activity were independent correlation factors based on binary logistic regression analyses (Table 3). To further confirm whether smoking history affected BChE activity in the two groups, a subgroup analysis was performed and is shown in Figure 1. There was no significant difference in serum BChE activity between PD patients with and without smoking history. The ROC curve of serum BChE activity for detecting PD patients is presented in Figure 2(a) and the AUC for PD was 0.699. Based on the ROC curve and diagnostic value of the different cut-off points, the optimal cut-off point, where the sensitivity value plus the specificity value was maximum, was found to be 6864.08 IU/L. The sensitivity and specificity values of serum BChE activity for detecting PD were 61.8% and 72.1%, respectively.

### 3.2. Disease Severity of PD Patients

For PD patients, serum BChE activity inversely correlated with UPDRS scores (*r* = −0.404, *P* < 0.001). Relationship between BChE activity and UPDRS score is presented in Figure 2(b).

### 3.3. Clinical Correlation Factors for BChE Activity in PD Patients

Results of univariate and multivariate linear regression analysis for serum BChE activity in PD patients are shown in Table 4. Gender, albumin, BMI, and dementia were all independent correlation factors for serum BChE activity. BChE activity positively correlated with MMSE score (*r* = 0.359, *P* = 0.001) and the relationship is presented in Figure 2(c). The medication status of these patients is presented in Table 5, and no significant drug influence can be found on serum BChE activity based on the univariate linear regression analyses.

### 3.4. BChE Activity in PD-Related Dementia

PD patients were categorized into two subgroups (with dementia and without dementia) per MMSE scores and education background. Clinical characteristics of these two subgroups are shown in Table 6. The table indicates that patients with dementia were older, had significantly longer disease duration, and presented with a more advanced stage. Patients with dementia had lower BChE activity than patients without dementia. However, according to binary logistic regression analysis, only advanced stage and BChE activity were independent correlation factors for PD-related dementia (Table 7). The ROC curve of serum BChE activity for patients with dementia is shown in Figure 2(d), and the AUC for patients with dementia was 0.752. Based on the ROC, the sensitivity and specificity values of serum BChE activity for diagnosing PD-related dementia were 70.6% and 76.3%, respectively, where the optimal cut-off point was 6550.00 IU/L.

## 4. Discussion

Ach is the main parasympathetic neurotransmitter and can be hydrolyzed by AChE and BChE, two enzymes in the circulation system. BChE accounts for up to 90% total serum ChE while its activity is 20-fold lower than AChE in hydrolyzing Ach [16]. Serum BChE activity has been broadly used as a biomarker for various diseases [17, 18], although its function has not been definitely clarified. In the clinic, it is most widely used for the assessment of organophosphate poisoning [8]. Organophosphates, as well as other forms of environmental toxin exposure, are risk factors for Parkinson's disease [9]. Serum BChE activity in Parkinson's disease has not yet been clearly determined, and we report it for the first time.

Results from this study showed significantly less BChE activity in PD patients compared with healthy controls while the other clinical characteristics showed no significant differences, suggesting the comparison was reasonable. PD patients usually have a number of comorbidities and BChE activity is reportedly related to many pathologies, including hepatic function [17] and metabolic syndrome (diabetes mellitus, hypercholesterolemia, TC, and TG) [11, 19]. Hence, linear regression analyses were performed, and PD was also found to be an independent correlation factor for BChE activity in our study, along with gender, albumin, TG, and BMI [11]. Binary logistic regression analyses were also performed for PD and determined smoking history and BChE activity as the independent correlation factors. Meanwhile, PD patients had a slightly lower percentage of smoking history compared with healthy controls, which was consistent with a former study [20]. Then a subgroup analysis found no alterations between patients with and without smoking history, suggesting that only BChE activity correlated with Parkinson's disease independent of hepatic function, smoking history, metabolic syndrome, or any other clinical characteristics.

However, the declined BChE activity was inconsistent with a former study [9], which indicated that BChE activity was not significantly altered in Israeli Parkinson's disease patients compared with younger healthy controls. Their result was controversial as some researchers showed an age-dependant difference in BChE activity [11, 21], which was also confirmed from our study. Furthermore, serum BChE activity had reportedly decreased in the organophosphates exposed population [22, 23]. However, nearly 37.6% of their PD patients were exposed to relatively high levels of organophosphates but without a decreased BChE activity. The inconformity may result from various genetic backgrounds of PD patients in different races. The AUC of ROC for Parkinson's disease in our study was 0.699, and the sensitivity and specificity values of serum BChE activity for diagnosing PD were 61.8% and 72.1%, respectively, with an optimal cut-off point of 6864.08 IU/L.

UPDRS consists of four assessments and is the most widely used clinical rating scale for evaluating disease severity in Parkinson's disease [24]. We found that BChE activity inversely correlated with UPDRS scores, suggesting that it could serve as a biomarker for evaluating disease severity in PD patient. Mini-Mental State Examination is the most popular scale to determine possible dementia and a positive correlation was also found between serum BChE activity and MMSE score.

In PD patients, we found that dementia as well as gender, albumin, and BMI, was an independent correlation factor for BChE activity. As all the patients were treated with dopamine analogues or dopamine receptor agonists at the time of enrolment, linear regression analyses were performed to exclude medication influence on BChE activity. Meanwhile, no significant results can be found. Results also showed that PD with dementia had a longer disease duration than PD without dementia, with no significant difference in onset age between these two groups, which was likely due to the face that dementia developed in most PD patients after a long disease duration [3]. These patients, therefore, were at a more advanced stage with more severe clinical symptoms. Additionally, PD patients with dementia had more difficulties in activities of daily living and required more assistance. Thus, their nutritional state was poorer, and BChE activity was lower than patients without dementia. Logistic regression analysis then confirmed that both BChE activity and advanced stage were independent correlation factors for PD-related dementia.

The ROC curve was analyzed for serum BChE activity to detect patients with dementia. The sensitivity and specificity values were 70.6% and 76.3%, respectively, with an optimal cut-off point of 6550.00 IU/L. The specificity value was high enough to discriminate between PD with dementia and patients without dementia.

In conclusion, BChE activity can be regarded as a biomarker in PD, especially in PD-related dementia. The same function has also been reported in other disorders, including Alzheimer's disease [11], vascular dementia [25], ischemic stroke [26], traumatic brain injury [7], postoperative delirium [27], severe trauma [28], septic shock [29], burn injuries [30], adverse cardiac events [16], hepatic disease [17], diabetes [31], and hyperlipidemia [32]. A former study on AChE levels in PD patients suggested that polymorphisms in* ACHE* genes fail to initiate ACHE expression, resulting in an impaired Ach/DA balance and subsequent vulnerability of dopaminergic neurons and PD development [9]. The Ach/DA imbalance seemed to have difficulty in explaining the alteration of BChE activity in the other disorders. Some studies have also suggested that BChE activity reflects the intensity of cholinergic anti-inflammatory responses [7] or serves as a marker of changes in the sympathetic/parasympathetic balance [33]. The authors were also reluctant to explain the extensive involvement of BChE activity in multisystem disorders. BChE activity is repeatedly reported to be positively associated with TG, TC, BMI, and metabolic syndrome and is inversely associated with malnutrition [11], which can be also confirmed from our results. BChE is mainly synthesized in the liver and then released into the blood, suggesting that it could serve as a “nutrition protein.” Levels decline with malnutrition due to disease or increase following overnutrition in metabolic syndrome. The potential pathophysiological mechanisms require further clarification. However, BChE activity could serve as a biomarker in PD patient to assess disease severity, discriminate PD-related dementia, evaluate efficacy of drugs, and instruct physicians about the course of clinical medications for dementia.

There were several limitations to this study. First, the number of included patients was limited and these results require further confirmation. This cohort study will continue and we hope it can also be validated by other research groups. Second, only BChE activity, without AChE activity, had been determined, as BChE activity was simple and convenient to obtain in our country.

## 5. Conclusions

Serum BChE activity, which decreased in PD patients and inversely correlated with disease severity, was clearly reported in PD patients for the first time. BChE activity could serve as a biomarker for detecting PD and related dementia in clinic.

## Figures and Tables

**Table 1:** Clinical characteristics of PD patients and healthy controls.

Variable (SD/%)	PD (76)	Control (61)	*P* value
Age (year)	69.30 ± 8.99	67.87 ± 5.74	0.260
Gender, male	45 (59.2%)	29 (47.5%)	0.173
Albumin (g/L)	38.99 ± 3.93	39.92 ± 3.52	0.152
ALT (U/L)	18.99 ± 13.58	20.03 ± 9.94	0.616
AST (U/L)	23.04 ± 13.59	20.93 ± 6.51	0.238
TC (mmol/L)	4.25 ± 0.84	4.19 ± 0.91	0.701
TG (mmol/L)	1.25 ± 0.76	1.45 ± 0.79	0.116
BMI	23.28 ± 3.36	23.71 ± 3.16	0.449
Smoking history	14 (18.2%)	20 (32.8%)	0.053
Alcohol consumption	7 (9.2%)	5 (7.8%)	0.835
CHD	15 (19.7%)	7 (10.9%)	0.191
Hypertension	29 (38.2%)	29 (47.5%)	0.269
Diabetes mellitus	10 (13.2%)	10 (16.4%)	0.594
Hypercholesterolaemia	15 (19.7%)	9 (14.8%)	0.446
BChE activity (IU/L)	6869.94 ± 1343.51	7904.29 ± 1470.77	<0.001

PD, Parkinson's disease; SD, standard deviation; ALT, alanine transaminase; AST, aspartate aminotransferase; TC, total cholesterol; TG, triglyceride; BMI, body mass index; CHD, coronary heart disease; BChE, butyrylcholinesterase.

**Table 2:** Univariate and multivariate linear regression analysis of correlation factors for serum butyrylcholinesterase activity.

Variable	Univariate		Multivariate	
	Beta value	*P* value	Beta value	*P* value
Age	−0.264	0.002	—	—
Gender	0.258	0.002	0.183	0.006
Albumin	0.477	0.000	0.350	0.000
ALT	−0.014	0.867	/	/
AST	−2.052	0.042	—	—
TC	3.876	0.000	—	—
TG	0.439	0.000	0.235	0.001
BMI	0.322	0.000	0.163	0.020
Smoking history	−0.028	0.744	/	/
Alcohol consumption	−0.001	0.991	/	/
Hypertension	0.074	0.392	/	/
Diabetes mellitus	0.059	0.496	/	/
Hypercholesterolaemia	0.166	0.052	—	—
CHD	−0.090	0.297	/	/
PD	−0.347	0.000	−0.240	0.000

Beta value, adjusted regression coefficient; ALT, alanine transaminase; AST, aspartate aminotransferase; TC, total cholesterol; TG, triglyceride; BMI, body mass index; CHD, coronary heart disease; PD, Parkinson's disease.

For the multivariate linear regression analysis using a backward method based on the results from univariate linear regression analysis, the values were “/” if variables were excluded before the analysis, and the values were “—” if variables were excluded after the analysis.

**Table 3:** Univariate and multivariate binary logistic regression analysis of correlation factors for Parkinson's disease.

Variable	Univariate		Multivariate	
	OR (LL~UL)	*P* value	OR (LL~UL)	*P* value
Age	1.025 (0.980~1.071)	0.281	/	/
Gender	0.624 (0.316~1.232)	0.174	/	/
Albumin	0.935 (0.852~1.026)	0.154	/	/
ALT	1.004 (0.988~1.021)	0.622	/	/
AST	1.022 (0.990~1.054)	0.175	/	/
TC	1.085 (0.718~1.639)	0.699	/	/
TG	0.700 (0.445~1.100)	0.121	/	/
BMI	0.960 (0.866~1.066)	0.446	/	/
Smoking history	0.463 (0.210~1.019)	0.056	0.404 (0.175~0.936)	0.034
Alcohol consumption	0.414 (0.152~1.128)	0.085	/	/
Hypertension	0.681 (0.344~1.348)	0.270	/	/
Diabetes mellitus	0.773 (0.299~1.997)	0.595	/	/
Hypercholesterolaemia	1.421 (0.575~3.513)	0.447	/	/
CHD	1.897 (0.720~4.999)	0.195	/	/
BChE activity	0.999 (0.999~1.000)	0.000	0.999 (0.999~1.000)	0.000

OR, odds ratio; LL, lower limit; UL, upper limit; ALT, alanine transaminase; AST, aspartate aminotransferase; TC, total cholesterol; TG, triglyceride; BMI, body mass index; CHD, coronary heart disease; BChE, butyrylcholinesterase.

For the multivariate binary logistic regression analysis using a conditional backward method based on the results from univariate binary logistic regression analysis, the values were “/” if variables were excluded before the analysis.

**Table 4:** Univariate and multivariate linear regression analysis of correlation factors for serum butyrylcholinesterase activity in patient with Parkinson's disease.

Variable	Univariate		Multivariate	
	Beta value	*P* value	Beta value	*P* value
Disease duration	−0.202	0.080	/	*/*
Age	−0.354	0.002	—	—
Gender	0.248	0.031	0.277	0.001
Albumin	0.568	0.000	0.493	0.000
ALT	0.029	0.802	/	/
AST	−0.196	0.089	/	/
TC	0.399	0.000	—	—
TG	0.261	0.023	—	—
BMI	0.305	0.007	0.188	0.029
Smoking history	0.001	0.993	/	/
Alcohol consumption	−0.051	0.662	/	/
Hypertension	0.024	0.834	/	/
Diabetes mellitus	0.076	0.515	/	/
Hypercholesterolaemia	0.133	0.251	/	/
CHD	−0.097	0.406	/	/
Advanced stage	−0.190	0.101	/	/
Dementia	−0.360	0.001	−0.319	0.000

Beta value, adjusted regression coefficient; ALT, alanine transaminase; AST, aspartate aminotransferase; TC, total cholesterol; TG, triglyceride; BMI, body mass index; CHD, coronary heart disease; PD, Parkinson's disease.

For the multivariate linear regression analysis using a backward method based on the results from univariate linear regression analysis, the values were “/” if variables were excluded before the analysis, and the values were “—” if variables were excluded after the analysis.

**Table 5:** Univariate linear regression analysis of anti-Parkinson's disease drugs for serum butyrylcholinesterase activity in PD patient.

Variable	Patient percentage	Beta value	*P* value
Total patients	76 (100%)	/	*/*
Levodopa and Benserazide Hydrochloride Tablet	54 (71.1%)	−0.169	0.143
Carbidopa and Levodopa-CR Tablet	8 (10.5%)	−0.106	0.362
Levodopa Tablet	1 (1.3%)	0.042	0.717
Piribedil-SR Tablet	27 (35.5%)	0.759	0.450
Pramipexole Dihydrochloride Tablet	15 (19.7%)	−0.042	0.720

PD, Parkinson's disease; Beta value, adjusted regression coefficient; CR, controlled release; SR, sustained release.

**Table 6:** Clinical characteristics of PD patients with and without dementia.

Variable (SD)	With dementia (17)	Without dementia (59)	*P*value
Disease duration (year)	8.71 ± 6.06	5.10 ± 4.23	0.032
Age (year)	73.47 ± 7.89	68.10 ± 8.98	0.029
Onset age (year)	64.76 ± 12.18	63.00 ± 9.28	0.523
Gender, male	9 (52.9%)	36 (61.0%)	0.551
Albumin (g/L)	38.18 ± 3.91	39.22 ± 3.94	0.338
ALT (U/L)	15.63 ± 10.74	19.90 ± 14.18	0.267
AST (U/L)	23.69 ± 19.87	22.86 ± 11.55	0.832
TC (mmol/L)	4.05 ± 0.77	4.31 ± 0.85	0.272
TG (mmol/L)	1.13 ± 0.75	1.27 ± 0.76	0.502
Smoking history	4 (23.5%)	10 (16.9%)	0.794
Alcohol consumption	3 (17.6%)	4 (6.8%)	0.374
Hypertension	5 (29.4%)	24 (40.7%)	0.399
Diabetes mellitus	2 (11.8%)	8 (13.6%)	1.000
Hypercholesterolaemia	2 (11.8%)	13 (22.0%)	0.554
CHD	6 (35.3%)	9 (15.3%)	0.138
BMI	23.12 ± 3.13	23.33 ± 3.45	0.822
UPDRS score	76.06 ± 16.80	48.36 ± 23.10	<0.001
Advanced stage	15 (88.2%)	30 (50.8%)	0.006
BChE activity (IU/L)	5974.96 ± 1292.90	7127.81 ± 1253.21	0.003

PD, Parkinson's disease; SD, standard deviation; ALT, alanine transaminase; AST, aspartate aminotransferase; TC, total cholesterol; TG, triglyceride; CHD, coronary heart disease; BMI, body mass index; UPDRS, unified Parkinson's disease rating scale; BChE, butyrylcholinesterase.

**Table 7:** Univariate and multivariate binary logistic regression analysis of correlation factors for Parkinson's disease related dementia.

Variable	Univariate		Multivariate	
	OR (LL~UL)	*P* value	OR (LL~UL)	*P* value
Disease duration	1.154 (1.033~1.290)	0.011	—	*—*
Age	1.080 (1.006~1.159)	0.034	—	—
Gender	1.391 (0.469~4.124)	0.551	/	/
Albumin	0.936 (0.817~1.071)	0.336	/	/
ALT	1.009 (0.992~1.026)	0.302	/	/
AST	1.018 (0.991~1.046)	0.197	/	/
TC	0.680 (0.343~1.349)	0.270	/	/
TG	0.758 (0.339~1.694)	0.499	/	/
BMI	0.981 (0.834~1.154)	0.820	/	/
Smoking history	1.508 (0.406~5.593)	0.539	/	/
Alcohol consumption	2.946 (0.590~14.708)	0.188	/	/
Hypertension	0.608 (0.189~1.949)	0.402	/	/
Diabetes mellitus	0.850 (0.163~4.439)	0.847	/	/
Hypercholesterolaemia	0.472 (0.095~2.334)	0.357	/	/
CHD	3.030 (0.893~10.284)	0.075	/	/
Advanced stage	7.250 (1.522~34.543)	0.013	5.612 (1.132~27.827)	0.035
BChE activity	0.999 (0.999~1.000)	0.004	0.999 (0.999~1.000)	0.013

OR, odds ratio; LL, lower limit; UL, upper limit; ALT, alanine transaminase; AST, aspartate aminotransferase; TC, total cholesterol; TG, triglyceride; BMI, body mass index; CHD, coronary heart disease; BChE, butyrylcholinesterase.

For the multivariate binary logistic regression analysis using a conditional backward method based on the results from univariate binary logistic regression analysis, the values were “/” if variables were excluded before the analysis, and the values were “—” if variables were excluded after the analysis.
